# Effects of Six Types of Exercise Interventions on Inhibitory Control, Executive Function, and Gross Motor Skills in Children With ADHD: A Network Meta‐Analysis of 26 Randomized Controlled Trials

**DOI:** 10.1002/brb3.71069

**Published:** 2025-12-07

**Authors:** Juan Ouyang, Yimin Hu, Yi Xia, Yi Sheng

**Affiliations:** ^1^ School of Athletic Performance Shanghai University of Sport Shanghai China

**Keywords:** ADHD, children, executive function, gross motor skills, inhibitory control

## Abstract

**Background:**

Attention deficit hyperactivity disorder (ADHD) is a neurodevelopmental disorder characterized by inattention, hyperactivity, and impulsivity, which adversely affect academic performance and social functioning in children. Current treatment approaches, including medication and behavioral therapy, present certain limitations. In recent years, exercise therapy has attracted increasing attention as a non‐pharmacological intervention without adverse side effects. However, the efficacy of conventional exercise regimens remains limited. Consequently, to address this limitation, researchers have developed a range of innovative exercise‐based interventions—such as mind–dody movement (MBM), ball games (BG), artificial intelligence training (AI), aerobic training (AT), circuit training (CIR), and neurofeedback training (NFT)—which have demonstrated significant benefits in improving inhibitory control, executive function, and gross motor skills among children with ADHD. This study aims to analyze the effects of these innovative exercise interventions and to provide a foundation for optimizing exercise therapy for this population.

**Methods:**

We systematically searched six databases, including PubMed, Web of Science, China National Knowledge Infrastructure (CNKI), Embase, Scopus, and SPORT Discus (EBSCOhost), to screen 26 randomized controlled trials (RCTs) covering 1,276 children with ADHD. The results of the study focused on the key metrics of inhibitory control, executive function, and gross motor skills. We used the Review Manager 5.3 software to assess the methodological quality of the studies. Based on the results criteria, we provided an overall rating and level of evidence for each attribute. We performed network meta‐analysis (NMA) using Stata 15.0 software with the aim of assessing the relative effectiveness of different interventions and verifying the consistency of direct and indirect evidence.

**Results:**

The NMA revealed distinct patterns of domain‐specific effectiveness across interventions. For gross motor skills, BG (SMD = ‐2.08, 95% CI [–3.70, –0.47]) and MBM (SMD = –1.59, 95% CI [–2.20, –0.98]) demonstrated the largest effect sizes, with CIR, NFT, and AI also showing statistically significant benefits. In the domain of inhibitory control, MBM again showed the strongest effect (SMD = –2.26, 95% CI [–2.97, –1.55]), followed by AT and NFT. For executive function, only MBM and NFT achieved statistically significant improvements compared to the control. Notably, MBM emerged as the only intervention with consistent trans‐domain efficacy, showing significant benefits in inhibitory control, executive function, and gross motor skills.

**Conclusion:**

This NMA indicates that while six exercise interventions generally benefit children with ADHD, their effects exhibit distinct domain specificity. A key finding is that mind‐body exercise emerged as the most universal and effective intervention across all measures, demonstrating the strongest effects on inhibitory control and significant benefits in both executive function and gross motor skills. NFT also demonstrated broad utility, significantly improving executive function and inhibitory control. Other interventions, such as ball sports and AT, showed effects more confined to specific domains like gross motor skills or inhibitory control.NMA comparing six exercise therapies for improving inhibitory function, executive function, and gross motor development in children with ADHD.

## Graphical abstract

Network meta‐analysis comparing six exercise therapies for improving inhibitory function, executive function, and gross motor development in children with ADHD.

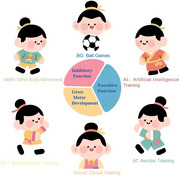

**Abbreviations**
ADHD: Attention Deficit Hyperactivity Disorder
AFT: Akergill Fatigue Test
AI: Artificial Intelligence Training
AT: Aerobic Training
BG: Ball Games
BOT‐2: Bruininks‐Oseretsky Test of Motor Proficiency‐Second Edition
BRIEF: Behavior Rating Inventory of Executive
CBCL: Child Behavior Checklist
CG: Control Group
Circuit: Circuit Training
CO‐D: WISC‐V‐Coding
CPT‐II: Conners’ Continuous Performance Test—Second Edition
CSC: Criterial Self‐Control
DAT: Deutscher Motornik Test
DISYPS‐II: Diagnostic System for Mental Disorders‐2
EG: Experimental Group
GMFM: Gross Motor Function Measure
M‐ABC‐2: Movement Assessment Battery for Children‐Second Edition TGMD
MBM: Mind‐Body Movement
MPST: Mirror Pursuit Steering Task
NFT: Neurofeedback Training
NMA: Network Meta‐Analysis
OST: Oddball Task
PDMS‐2: Peabody Developmental Motor Scales—Second Edition
RD: Reward Delay
SCWI: Stroop Color‐Word Interference
Stroop: Stroop Test
SWAN: Strengths and Weaknesses of ADHD Symptoms and Normal Behavior
WCST: Wisconsin Card Sorting Test

## 1 Introduction

ADHD is a neurodevelopmental disorder characterized by inattention, hyperactivity, and impulsivity, which significantly impair daily functioning and learning in children (Ashdown‐Franks et al. 2020; Cortese et al. 2015). Specifically, inattention contributes to poor academic performance and difficulties with task completion (Liang et al. 2022; Benzing and Schmidt 2019), whereas hyperactivity often disrupts classroom and home environments, thereby straining social relationships (Fang et al. 2024; Pan et al. 2016). Impulsivity can increase the risk of accidents and peer conflicts (Pontifex et al. 2013). Long‐term, ADHD exerts a negative impact on self‐esteem, social development, and overall quality of life (Wexler et al. 2021).

Inhibitory function, a core component of executive function, enables children to suppress distractions and maintain focus on tasks (Diamond 2013; Miyake et al. 2000). Deficits in EF hinder the ability to complete complex tasks and impede social adjustment (Zang 2019). Concurrently, gross motor development (e.g., running, jumping) is crucial for fostering physical health, self‐confidence, and peer interaction. Delays in this area may lead to physical clumsiness, social isolation, and low self‐esteem (Cairney et al. 2005; Skinner and Piek 2001). Children with ADHD frequently demonstrate motor coordination difficulties, which can further compromise their social integration and overall well‐being.

Current treatments for ADHD include medication, which is effective but often associated with side effects such as decreased appetite and sleep disturbances (Smith et al. 2020), and behavioral therapy, which shows variable efficacy and requires long‐term involvement from caregivers (Meßler et al. 2018). Exercise therapy has emerged as a non‐pharmacological alternative that is sustainable and free from adverse side effects, with the potential to enhance attention, behavior, motor skills, and self‐regulation in children with ADHD (Vysniauske et al. 2020).

In recent years, non‐pharmacological interventions for ADHD have gained considerable interest due to their favorable safety profile and multi‐faceted benefits. Exercise therapy is defined as a prescribed, planned, structured, and repetitive form of physical activity conducted under the guidance or supervision of qualified professionals. Unlike general physical fitness activities, it is designed as an individualized intervention aimed at improving or restoring physical function, promoting health, or managing specific symptoms related to chronic conditions or injuries (Caspersen et al. 1985, Pedersen and Saltin 2015).

This study focuses on six distinct exercise interventions for children with ADHD (Table 1). MBM integrates psychological regulation with physical activity to enhance the mind‐body connection through breath control and movement coordination, as seen in practices like yoga (Jensen and Kenny 2004). BG utilize sports such as soccer and basketball to improve executive function via rapid visual tracking, response inhibition, and decision‐making (Pan et al. 2016). AT consists of regular aerobic exercises, such as running or swimming, designed to enhance inhibitory control (Zhu et al. 2023). CIR employs a multi‐station model that combines aerobic, strength, and flexibility exercises, improving executive function through structured station rotation (Ghai et al. 2017). NFT is a structured neuromodulation intervention based on operant conditioning, involving planned, repetitive sessions where individuals learn to self‐regulate brainwave activity (e.g., modulating the theta/beta ratio) using real‐time electroencephalogram (EEG) feedback in a controlled setting (Gevensleben et al. 2009). A typical protocol may involve children completing three 30‐minute sessions per week over 10 weeks, using EEG to control a computer game by maintaining a focused state (Kollins et al. 2020). AI applies machine learning, real‐time data analysis, and adaptive interaction to personalize and enhance structured interventions. This includes AI‐driven exergames on platforms such as Xbox Kinect or Nintendo Wii, which adjust exercise difficulty and feedback based on user performance (Bustamante et al. 2022).

**TABLE 1:** Intervention methods.

Intervention methods	Abbreviation	Definition	Example
Mind‐body movement	MBM	an intervention that combines psychological regulation with physical movement, promoting mind‐body connection through breath control and movement coordinationb (Jensen and Kenny 2004).	yoga, tai chi
Ball games	BG	an intervention that uses sports such as soccer and basketball to improve the executive function of children with ADHD through rapid visual tracking, response inhibition, and decision adjustmentb (Pan et al. 2016).	soccer, basketball, racket sports
Aerobic training	AT	a regular aerobic exercise program, aimed at improving the inhibitory control of children with ADHD (Zhu et al. 2023).	running, swimming
Circuit training	CIR	a multi‐station training model combining aerobic, strength, and flexibility training, which enhances executive function in children with ADHD through station rotation (Ghai et al. 2017).	Circuit Training Protocol for Children with ADHD, Station 1: Jumping Jacks, Station 2: Medicine Ball Slams, Station 3: Spiderman Crawl,Station 4: Yoga—Warrior II Pose, Station 5: Agility Ladder Drills, Station 6: Bear Hold (Static)
Neurofeedback training	NFT	a structured neuromodulation intervention grounded in operant conditioning. It involves planned and repetitive sessions where individuals learn to self‐regulate their brainwave activity through real‐time feedback from electroencephalogram (EEG) in a clinical or laboratory setting (Gevensleben et al. 2009).	Theta/Beta Training
Artificial intelligence	AI	a methodological approach that employs machine learning, real‐time data analysis, and adaptive interaction to personalize and enhance structured interventions (Kollins et al. 2020).	AI‐enhanced exergames utilizing platforms like Xbox Kinect or Nintendo Wii

The selection and categorization of these six interventions were guided by two primary rationales to ensure a comprehensive and logically structured comparison. First, they represent a spectrum of distinct mechanisms of action—from mind‐body integration (MBM) and sport‐specific skill acquisition (BG) to cardiovascular exertion (AT), composite fitness training (CIR), and technology‐driven neuromodulation (NFT and AI). This allows for an examination of whether benefits for ADHD symptoms are mechanism‐specific. Second, this framework facilitates a critical comparison between established traditional exercise modalities (AT, BG, CIR, MBM) and emerging technology‐assisted interventions (NFT, AI), assessing whether innovative approaches offer superior advantages. It is noteworthy that our operational definition of “exercise intervention” is broad, centering on scheduled, repetitive regimens with a core physical component. This inclusive approach enables a principled comparison across the spectrum of active interventions, ensuring that our NMA can provide nuanced insights into the most effective types for improving specific cognitive and motor outcomes in children with ADHD.

A growing body of evidence suggests potential benefits of exercise interventions for core symptoms and cognitive deficits associated with ADHD (Zang 2019). However, findings are heterogeneous, and the effects of physical activity are known to vary based on intervention characteristics and individual factors (Tang et al. 2015). Consequently, while the overall promise is recognized, the differential effects of specific exercise types on distinct symptom domains remain unclear, and there is a pronounced lack of evidence directly comparing the efficacy of multiple interventions. Therefore, this study aims to systematically evaluate and compare the effects of these six exercise interventions on inhibitory control, executive function, and gross motor skills in children with ADHD using a NMA.

## 2 Methods

We conducted this study following the guidelines of the preferred reporting items for systematic evaluation and meta‐analyses (the PRISMA list for NMAs10 and the Cochrane handbook for the evaluation of intervention systems). Registration number: CRD42024625651.

### 2.1 Search Strategy

We conducted systematic searches through electronic databases PubMed, Web of Science, CNKI, EBSCOhost, and two researchers (YO, XL) independently completed the selection of included studies. Our search covered the period from the inception of each database to December 2024. We limited the search to human‐related and peer‐reviewed articles. The search strategy followed the PICOS principles:

(P) Population: children with ADHD (4–16 years old)
(I) Interventions: Resistance training, autogenic training, endurance training, high‐intensity interval training, exercise movement techniques, exercise, warm‐up exercise, plyometric exercise
(C) Control group: the control group has no therapy or other non‐invasive treatments
(O) Outcome: Inhibitory control (measuring tools: stroop color‐word test, go/no‐go task, stop‐signal task), executive function (measuring tools: Wisconsin card sorting BRIEF), and gross motor skills (movement assessment battery for children‐2 (MABC‐2), test of gross motor skills (TGMD‐3))
(S) Study type: RCTs. The reference lists of relevant articles were also manually screened for other studies that might be eligible. The search timeframe was from January 2004 to December 2024, and the search was limited to human studies published in Chinese or English.

### 2.2 Study Selection

**FIGURE 1:**
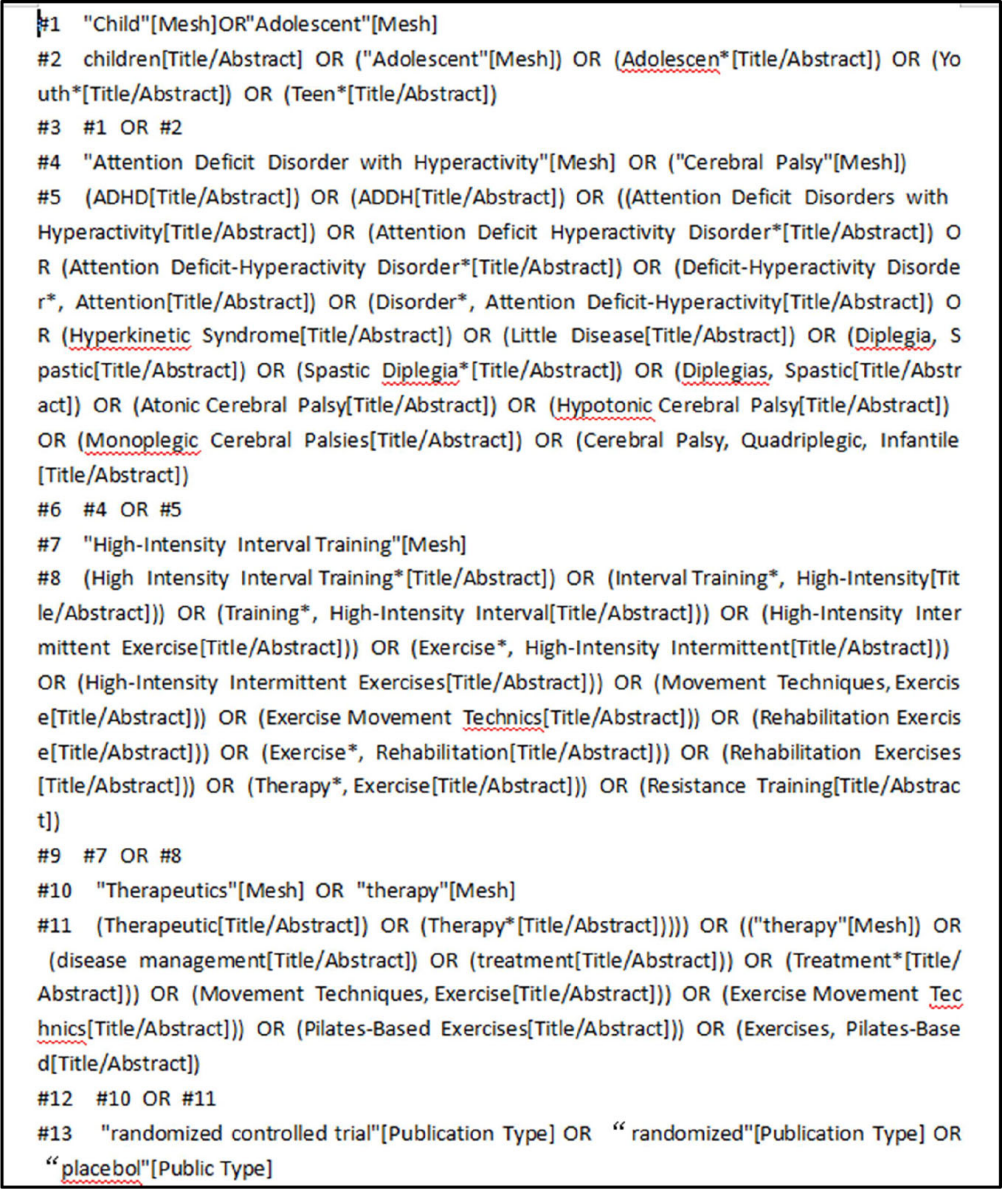
Literature search formula or literature search strategy.

Using the PubMed database as an example, in order to ensure greater access to literature related to sport, exercise, children with ADHD, and the development of gross motor skills, we conducted searches using multiple relevant search terms, including “exercise” [MeSH], “therapeutics” [MeSH], “treatment” [MeSH], “children” [MeSH], “gross motor skills” [MeSH], and “attention deficit disorders with hyperactivity” [MeSH]. Using these search terms, we were able to cover a wide range of research related to this field. See Figure 1 for specific search formulas.

A rigorous screening procedure was implemented to identify eligible studies. First, automated de‐duplication was performed using EndNote X9 software to remove duplicate records resulting from differing search strategies or data sources. Subsequently, a manual review of titles and abstracts was conducted to eliminate any remaining duplicates that the automated process might have missed, thereby ensuring the uniqueness and representativeness of the selected literature.

For the remaining articles, a more detailed evaluation was carried out. Studies were excluded based on the following criteria: those not involving a pediatric ADHD population, those not assessing relevant outcome indicators, and those not utilizing exercise‐based interventions. Additionally, we excluded review articles, conference abstracts, animal studies, research protocols, case reports, retrospective analyses, and book chapters, as these publications typically lack sufficient primary data or scientific rigor to support robust conclusions regarding intervention effects.

These stringent screening criteria were applied to ensure the high quality of the included studies, thereby establishing a solid evidence base for the analysis and enhancing the scientific validity and credibility of the present study (Figure 1).

### 2.3 Inclusion and Exclusion Criteria

We included studies if they

(1) were RCTs;
(2) enrolled children under 16 years of age with a confirmed diagnosis of ADHD
(3) implemented an exercise‐based intervention
(4) reported complete data for the outcome metrics
(5) assessed at least one of the following outcomes: gross motor skills, executive function, or inhibitory control; and
(6) were peer‐reviewed articles with the full text available.

We excluded studies if they

(1) were non‐RCTs
(2) were experimental animal studies, review literature, conference reports, case reports, letters, and repetitive publications
(3) were not available in full text
(4) had incomplete outcome data or data that could not be extracted or converted for analysis
(5) did not report outcomes relevant to this review; and
(6) involved patients with comorbid motor disorders that could confound the results.

### 2.4 Data Collection

Two researchers independently imported the retrieved literature into EndNote X9 software according to the predefined search strategy. The study selection process, including the number of records screened and those meeting the inclusion criteria, is summarized in Figure 1. To ensure the accuracy of the systematic screening process, duplicates were first removed using EndNote X9, followed by a preliminary screening based on titles and abstracts. The remaining articles underwent full‐text review against the predefined inclusion and exclusion criteria.

The two researchers cross‐checked their screening results. Studies for which both reviewers reached consensus were included. Any disagreements were resolved through discussion, and if no consensus could be reached, a third researcher (YH) was consulted to make the final decision. Two trained researchers independently extracted data from the included studies (see Table 2) and assessed the risk of bias (ROB) using a standardized data extraction form. The extracted data included (a) descriptive information (e.g., authors, publication year, country/region); (b) sample characteristics (e.g., age range, diagnostic method, sample size per group); (c) intervention characteristics (e.g., frequency, duration); and (d) measurement instruments used to assess inhibitory control, executive function, and gross motor skills.

**TABLE 2:** Basic features of the included studies.

Study	Country	Sample size (F/M)	Age (mean ± SD)	Intervention category	Intervention Modality	Intervention frequency and duration	Inhibitory control	Executive function	Gross motor
Jarraya 2019 (Jarraya et al. 2019)	Germany	15 (EG)	5.2 ± 0.4 (EG)	MBM (EG)	Yoga	2 days/week, 12 weeks	ADHD Rating Scale‐IV	—	DAT
		15 (CG)	5.2 ± 0.4 (CG)	No (CG)	—				
Haffner 2006 (Haffner et al. 2006)	Germany	8 (EG)	8.4 – 12.9 (EG)	MBM (EG)	Yoga	2 days/week, 8 weeks	Impulsivität (I)	Unaufmerksamkeit (U)	DAT
		8 (CG)	8.4 – 12.9 (CG)	No (CG)	—				
Saxena 2020 (Saxena et al. 2020)	USA	123 (74/49) (EG)	14.73 ± 0.41 (EG)	MBM (EG)	Yoga	2 days/week, 12 weeks	ADHD symptoms	SWAN	—
		51 (38/13) (CG)	14.84 ± 0.47 (CG)	No (CG)	—				
Luo 2023 (Luo et al. 2023)	China	15 (EG)	5.00 ± 0.76 (EG)	MBM (EG)	Yoga	2 days/week, 16 weeks	MTA SNAP‐IV (Inattention)	MTA SNAP‐IV (Hyperactivity‐Impulsivity)	—
		15 (CG)	5.07 ± 0.88 (CG)	No (CG)	—				
Salleg 2024 (Salleg‐Cabarcas et al. 2024)	Colombia	20 (EG)	10.5 ± 1.4 (EG)	NFT (EG)	Neurofeedback training	3 days/week, 12 weeks	CSC	RD	TGMD
		16 (CG)	10.5 ± 1.4 (CG)	No (CG)	—				
Pan 2016 (Pan et al. 2016)	China	16 (EG)	8.93 ± 1.49 (EG)	BG (EG)	Racquet sports	2 days/week, 12 weeks	Stroop	CBCL	OT‐2 (BOT‐2)
		16 (CG)	8.87 ± 1.56 (CG)	No (CG)	—				
Barkn 2023 (Barkin et al. 2023)	Turkey	88 (EG)	7 – 12 (EG)	AI (EG)	VR	2 days/week, 8 weeks	Stroop	—	OT‐2 (BOT‐2)
		88 (CG)	7 – 12 (CG)	No (CG)	—				
Tascioglu 2018 (Tascioglu et al. 2024)	Turkey	17 (EG)	10.1 ± 1.38 (EG)	CIR (EG)	High‐intensity interval training	2 days/week, 8 weeks	MPST	—	BOT‐2
		17 (CG)	9.68 ± 1.11 (CG)	No (CG)	—				
Meßler 2018 (Meßler et al. 2018)	Germany	14 (EG)	11 ± 1 (EG)	CIR (EG)	High‐Intensity Interval Training	3 days/week, 3 weeks	MPST	DISYPS‐II	M‐ABC‐2
		14 (CG)	11 ± 1 (CG)	No (CG)	—				
Ghadamgahi 2022 (Ghadamgahi Sani et al. 2022)	Iran	20 (EG)	7.8 ± 1.28 (EG)	NFT (EG)	Neurofeedback Training	3 days/week, 7 weeks	—	—	BOT‐2
		20 (CG)	7.5 ± 1.34 (CG)	PM exercises (CG)	—				
Benzing 2019 (Benzing and Schmidt 2019)	Switzerland	28 (EG)	10.46 ± 1.30 (EG)	AI (EG)	VR games	3 days/week, 8 weeks	Stroop	WCST	—
		23 (CG)	10.39 ± 1.44 (CG)	NO (CG)	—				
Chang 2022 (Chang et al. 2022)	China	16 (EG)	8.31 ± 1.30 (EG)	BG (EG)	Simulated ball	3 days/week, 12 weeks	Stroop	WCST	BOT‐2
		16 (CG)	8.31 ± 1.30 (CG)	NO (CG)	—				
Shuai 2021 (Shuai et al. 2021)	Netherlands	29 (EG)	7 – 13 (EG)	NFT (EG)	Neurofeedback Training	Day/week, 10 weeks	—	BRIEF	PDMS‐2
		25 (CG)	7‐13 (CG)	PA(CG)	—				
Liang 2022 (Liang et al. 2022)	China	40 (EG)	8.46 ± 1.50 (EG)	AT (EG)	Aerobic	3 days/week, 12 weeks	AFT	—	—
		40 (CG)	8.46 ± 1.50 (CG)	NO (CG)	—				
van 2014 (van der Oord et al. 2014)	Netherlands	40 (EG)	8 – 12 (EG)	NFT (EG)	Neurofeedback Training	5 days/week, 5 weeks	Stroop	BRIEF	PDMS‐2
		40 (CG)	8 – 12 (CG)	NO (CG)	—				
Lan 2020 (Lan et al. 2020)	China	40 (EG)	9 – 12 (EG)	NFT (EG)	Team Executive Function Training	day/week, 12 weeks	CPT‐II	OST	—
		40 (CG)	9 – 12 (CG)	No (CG)	—				
Chang 2012 (Chang et al. 2012)	China	20 (EG)	8 – 13 (EG)	AT (EG)	Running	2 days/week, 12 weeks	Stroop	WCST	BOT‐2
		20 (CG)	8 – 13 (CG)	NO (CG)	—				
Huang 2017 (Huang et al. 2017)	China	15 (EG)	7.93 ± 1.02 (EG)	AT (EG)	Aerobics	2 days/week, 8 weeks	Stroop	DISYPS‐II	PDMS‐2
		14 (CG)	8.27 ± 1.04 (CG)	NO (CG)	—				
Zhao 2024 (Zhao et al. 2024)	China	40 (EG)	8.5 ± 1.5 (EG)	NFT (EG)	Brain Training	3 days/week, 4 weeks	BRIEF	BRIEF	PDMS‐2
		40 (CG)	8.3 ± 1.1 (CG)	NO (CG)	—				
Liang 2024 (Liang et al. 2022)	China	15 (EG)	8.62 ± 1.37 (EG)	AT (EG)	Aerobic	3 days/week, 12 weeks	—	CO‐D	—
		15 (CG)	8.82 ± 1.59 (CG)	NO (CG)	—				
Janssen 2016 (Janssen et al. 2016)	Netherlands	29 (EG)	7 – 13 (EG)	NFT (EG)	Neurofeedback Training	day/week, 10 weeks	CPT‐II	—	PDMS‐2
		25 (CG)	7 – 13 (CG)	NO (CG)	—				
Cheng 2016 (Chen and Cheng 2016)	China	18 (8/10) (EG)	8.48 ± 0.52 (EG)	MBM (EG)	Tai Chi	3 days/week, 16 weeks	MPST	WCST	BOT‐2
		18 (9/9) (CG)	8.51 ± 0.5 (CG)	NO (CG)	—				
Meng 2004 (Meng et al. 2004)	China	20 (EG)	8 – 9 (EG)	MBM (EG)	Tai Chi	2 days/week, 10 weeks	SCWI	WCST	—
		13 (CG)	8 – 9 (CG)	NO (CG)	—				
Kadri 2019 (Kadri et al. 2019)	Manouba	20 (EG)	14.5 ± 3.5 (EG)	MBM (EG)	Taekwondo	2 days/week, 78 weeks	—	—	PDMS‐2
		20 (CG)	14.2 ± 3 (CG)	NO (CG)	—				
Vahid 2023 (Nejati et al. 2023)	Iran	15 (EG)	9.07 ± 3.40 (EG)	NFT (EG)	Neurofeedback training	4 weeks	Go/No‐Go Test	WCST	GMFM
		15 (CG)	9.07 ± 3.40 (CG)	NO (CG)					
Leanne 2013 (Tamm et al. 2013)	USA	54 (EG)	9.1 ± 1.2 (EG)	NFT (EG)	Neurofeedback training	2 days/week, 8 weeks	TEA‐Ch	BRIEF	GMFM
		51 (CG)	9.5 ± 1.5 (CG)	NO (CG)					

### 2.5 ROB of the Systematic Review

According to the Cochrane 5.1 version of the ROB assessment tool, which includes six domains (random sequence generation, allocation concealment, blinding of outcome assessor, incomplete data outcome, selective reporting, and other bias), two researchers (YO, XL) assessed the ROB for all eligible studies. We performed risk assessment analyses using Review Manager 5.3 (Nordic Cochrane, Denmark). Within each domain, the results were categorized as “unclear”, “low risk”, and “high risk”. Low ROB: No domains were assessed as high risk; there may be domains assessed as unclear, but no more than two. Medium ROB: There is one area assessed as high risk, or no areas assessed as high risk, but more than two areas assessed as unclear. High ROB: All other scenarios other than the above are categorized as high risk.

### 2.6 Statistical Analysis

We conducted the NMA using Stata 15.0 software (StataCorp LLC, College Station, TX, USA), treating all outcomes as continuous variables. The effects of various exercise interventions on inhibitory control, executive function, and gross motor skills in children with ADHD were evaluated by synthesizing pre‐post change data from both experimental and control groups. To accurately estimate intervention effects, we calculated standardized mean differences (SMDs) and their 95% confidence intervals (CIs) for each outcome, with the significance level uniformly set at α = 0.05.

A random‐effects model was employed to combine effect estimates, accounting for anticipated heterogeneity across studies in participant characteristics and intervention protocols. Between‐study heterogeneity was quantified using the *I^2^* statistic and Cochran's Q test.

To illustrate the relationships among different exercise modalities, we constructed a network diagram where nodes represent interventions and connecting lines indicate direct comparisons. The size of each node and the thickness of each line were proportional to the number of studies included for that comparison, visually representing the relative strength, and position of each intervention within the network. We further generated a network contribution plot to quantify how much each direct comparison contributed to the overall network estimates, thereby clarifying the influence of individual interventions.

Publication bias for key outcomes was assessed using adjusted comparison funnel plots. Finally, the probability of each intervention being the most effective was estimated using the surface under the cumulative ranking curve (SUCRA) method. This comprehensive approach allowed for a thorough evaluation of the relative efficacy of different exercise modalities for children with ADHD.

## 3 Result

### 3.1 Study Selection

**FIGURE 2:**
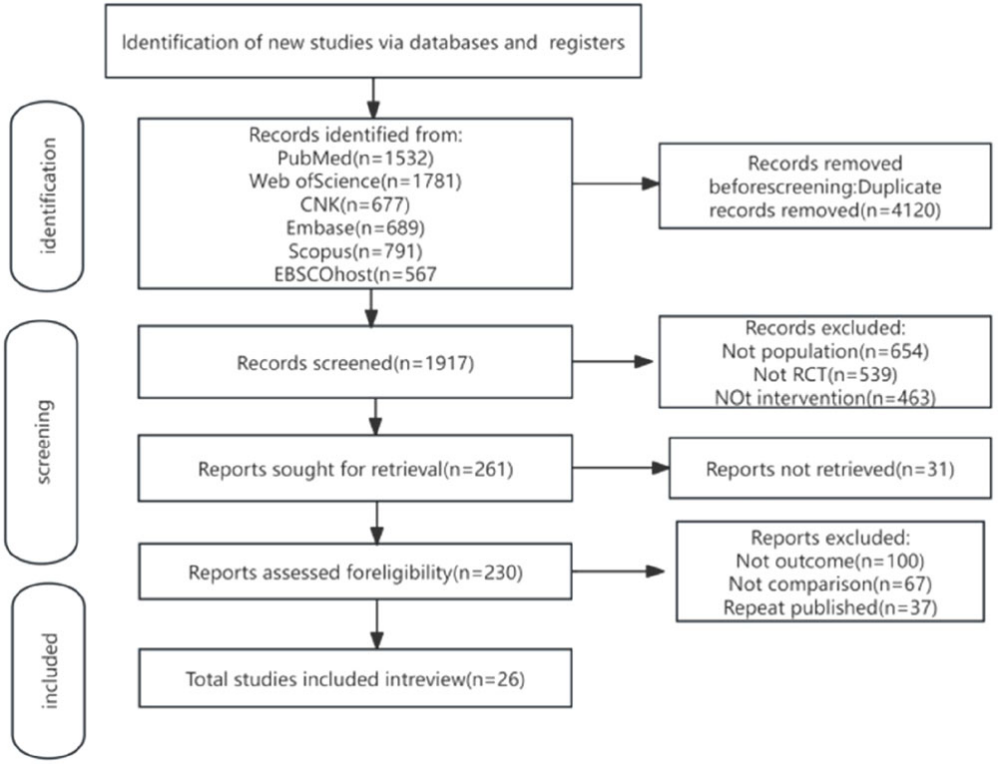
Study selection flowchart.

The flowchart of the study selection is presented in Figure 2. A total of 6,037 potentially eligible articles were identified from six databases: PubMed (*n* = 1532), Web of Science (*n* = 1781), CNKI (*n* = 677), Embase (*n* = 689), Scopus (*n* = 791), and SPORT Discus (EBSCOhost) (*n* = 567). After the removal of 4120 duplicates through automated and manual checking, 1917 records underwent title and abstract screening. This resulted in the exclusion of 1656 ineligible records (e.g., non‐RCTs, irrelevant interventions, or off‐target populations). The full texts of the remaining 230 articles were retrieved and thoroughly assessed for study design, sample size, methodological quality, and results. Ultimately, 26 studies met all predefined inclusion and quality criteria. This standardized selection process ensured the reliability and scientific validity of the review.

### 3.2 Characteristics of Included Studies

The systematic review included 26 RCTs, with their characteristics detailed in Table 2. Conducted between 2004 and 2024 across nine countries (e.g., China, Iran, Germany, and the USA), these studies involved a total of 1276 children and adolescents with ADHD. Reported demographic data consisted of country, age, and gender. The relevant outcome measures selected for this review were those pertaining to inhibitory control, executive function, and gross motor skills.

### 3.3 ROB Assessment

**FIGURE 3:**
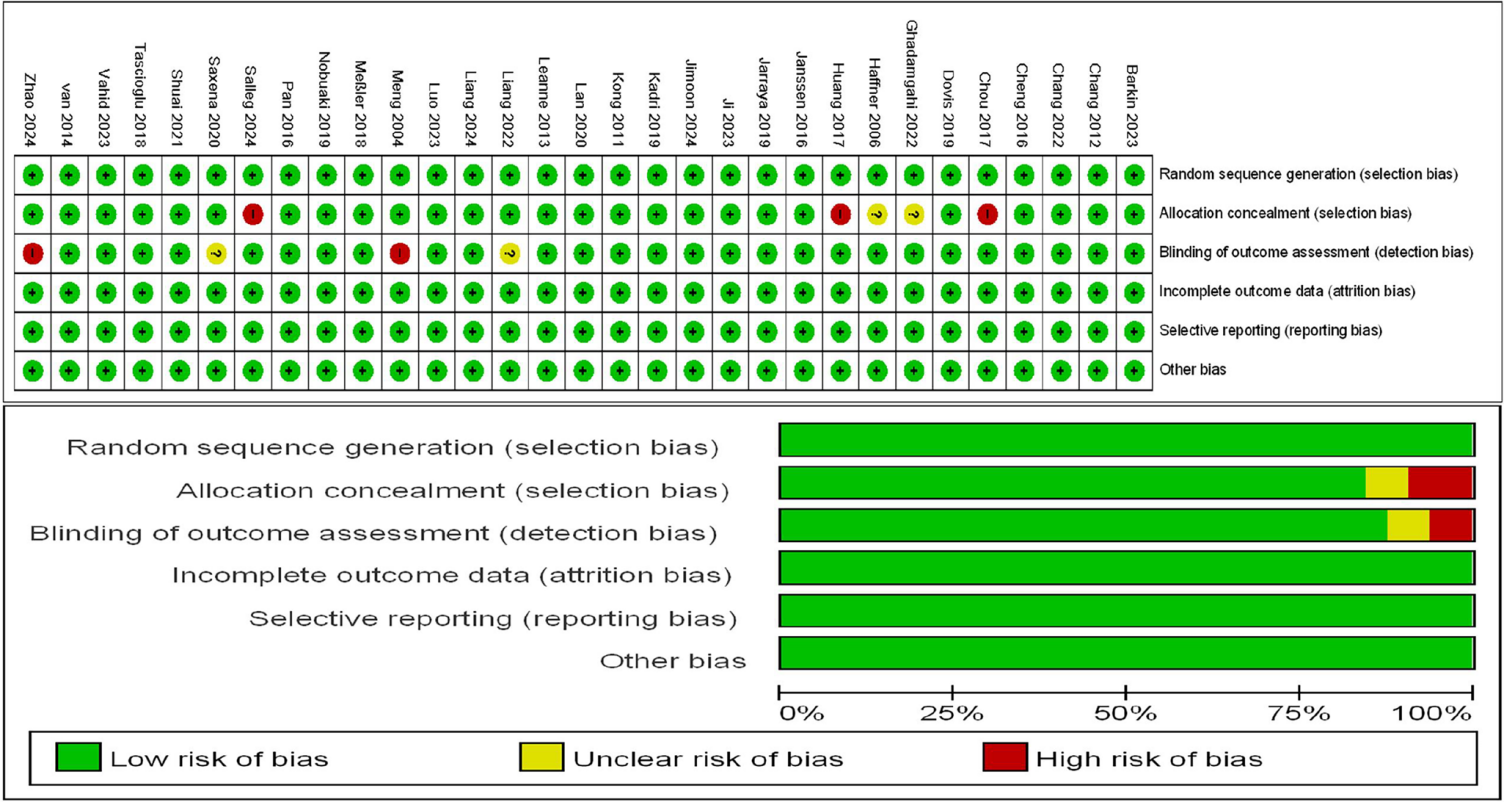
ROB assessment for included studies.

The ROB assessment is shown in Figure 3, detailing the results of 26 studies in the area of ROB. 26 articles mentioned random allocation; 21 stated allocation concealment; 22 reported blinding of outcome assessment; 26 studies showed a low risk of selective reporting; and 26 had no other bias. In summary, 23 articles were judged to have a low ROB.

### 3.4 Forest Plots From the Pairwise Meta‐Analyses for Outcomes

The forest plot results represent direct comparisons, where direct evidence originates from studies that directly compare the treatments of interest (e.g., A vs B) (Caldwell et al. 2005), It should be noted that these pairwise comparisons inform on the relative efficacy between two specific interventions but do not reflect the overall ranking of all interventions within the network. However, the effectiveness comparison of different interventions requires the meta‐analysis matrix of outcomes (Table 3), as it integrates both direct and indirect comparison resultsthe relative efficacy betweNMA. Indirect evidence comes from comparing two treatments through their relative effects on a common comparator (e.g., A vs. C and B vs. C provides indirect evidence about A vs. B) (Caldwell et al. 2005). Therefore, the final ranking of intervention measures in this report regarding statistical significance and effect size is based on the NMA results in Table 3. The forest plot data, as a presentation of direct evidence, has certain limitations, which we have explained in the section discussing limitations. It should be noted that these pairwise comparisons inform on the relative efficacy between two specific interventions but do not reflect the overall ranking of all interventions within the network.

**TABLE 3:** NMA matrix of outcome.

Inhibitory control						
MBM	1.04 (–0.21, 2.29)	1.18 (–0.29, 2.65)	1.35 (–0.58, 3.28)	1.41 (0.44, 2.37)	1.71 (–0.21, 3.64)	2.26 (1.55, 2.97)
−1.04 (–2.29, 0.21)	AT	0.14 (–1.50, 1.78)	0.31 (–1.76, 2.38)	0.37 (–0.85, 1.58)	0.67 (–1.38, 2.73)	1.22 (0.20, 2.24)
−1.18 (–2.65, 0.29)	−0.14 (–1.78, 1.50)	CIR	0.17 (–2.03, 2.38)	0.23 (–1.21, 1.67)	0.54 (–1.66, 2.73)	1.08 (–0.20, 2.36)
−1.35 (–3.28, 0.58)	−0.31 (–2.38, 1.76)	−0.17 (–2.38, 2.03)	AI	0.06 (–1.85, 1.97)	0.36 (–2.17, 2.90)	0.91 (–0.89, 2.71)
−1.41 (–2.37, –0.44)	−0.37 (–1.58, 0.85)	−0.23 (–1.67, 1.21)	−0.06 (–1.97, 1.85)	NFT	0.31 (–1.60, 2.21)	0.85 (0.20, 1.50)
−1.71 (–3.64, 0.21)	−0.67 (–2.73, 1.38)	−0.54 (–2.73, 1.66)	−0.36 (–2.90, 2.17)	−0.31 (–2.21, 1.60)	BG	0.54 (–1.24, 2.33)
−2.26 (–2.97, –1.55)	−1.22 (–2.24, –0.20)	−1.08 (–2.36, 0.20)	−0.91 (–2.71, 0.89)	−0.85 (–1.50, –0.20)	−0.54 (–2.33, 1.24)	CON
Executive function						
MBM	0.55 (–0.59, 1.69)	0.69 (–0.63, 2.01)	0.77 (–1.27, 2.80)	0.86 (–0.47, 2.18)	1.23 (–0.80, 3.26)	1.68 (0.88, 2.47)
−0.55 (–1.69, 0.59)	NFT	0.14 (–1.20, 1.47)	0.21 (–1.84, 2.26)	0.30 (–1.04, 1.64)	0.67 (–1.36, 2.71)	1.12 (0.30, 1.94)
−0.69 (–2.01, 0.63)	−0.14 (–1.47, 1.20)	AI	0.07 (–2.08, 2.23)	0.16 (–1.34, 1.66)	0.54 (–1.61, 2.68)	0.98 (–0.07, 2.04)
−0.77 (–2.80, 1.27)	−0.21 (–2.26, 1.84)	−0.07 (–2.23, 2.08)	CIR	0.09 (–2.07, 2.25)	0.46 (–2.18, 3.11)	0.91 (–0.97, 2.79)
−0.86 (–2.18, 0.47)	−0.30 (–1.64, 1.04)	−0.16 (–1.66, 1.34)	−0.09 (–2.25, 2.07)	AT	0.37 (–1.78, 2.52)	0.82 (–0.24, 1.88)
−1.23 (–3.26, 0.80)	−0.67 (–2.71, 1.36)	−0.54 (–2.68, 1.61)	−0.46 (–3.11, 2.18)	−0.37 (–2.52, 1.78)	BG	0.45 (–1.42, 2.32)
−1.68 (–2.47, –0.88)	−1.12 (–1.94, –0.30)	−0.98 (–2.04, 0.07)	−0.91 (–2.79, 0.97)	−0.82 (–1.88, 0.24)	−0.45 (–2.32, 1.42)	CON
Gross motor skills						
BG	0.49 (–1.23, 2.22)	0.88 (–1.07, 2.83)	0.88 (–0.94, 2.71)	1.41 (–0.41, 3.23)	1.53 (–0.17, 3.23)	2.08 (0.47, 3.70)
−0.49 (–2.22, 1.23)	MBM	0.38 (–0.88, 1.65)	0.39 (–0.65, 1.44)	0.92 (–0.13, 1.97)	1.04 (0.22, 1.85)	1.59 (0.98, 2.20)
−0.88 (–2.83, 1.07)	−0.38 (–1.65, 0.88)	CIR	0.01 (–1.39, 1.40)	0.53 (–0.86, 1.93)	0.65 (–0.58, 1.88)	1.21 (0.10, 2.31)
−0.88 (–2.71, 0.94)	−0.39 (–1.44, 0.65)	−0.01 (–1.40, 1.39)	AI	0.52 (–0.68, 1.73)	0.64 (–0.37, 1.65)	1.20 (0.35, 2.05)
−1.41 (–3.23, 0.41)	−0.92 (–1.97, 0.13)	−0.53 (–1.93, 0.86)	−0.52 (–1.73, 0.68)	AT	0.12 (–0.89, 1.13)	0.68 (–0.18, 1.53)
−1.53 (–3.23, 0.17)	−1.04 (–1.85, –0.22)	−0.65 (–1.88, 0.58)	−0.64 (–1.65, 0.37)	−0.12 (–1.13, 0.89)	NFT	0.55 (0.01, 1.10)
−2.08 (–3.70, –0.47)	−1.59 (–2.20, –0.98)	−1.21 (2.31, –0.10)	−1.20 (–2.05, –0.35)	−0.68 (–1.53, 0.18)	−0.55 (–1.10, –0.01)	CON

**FIGURE 4:**
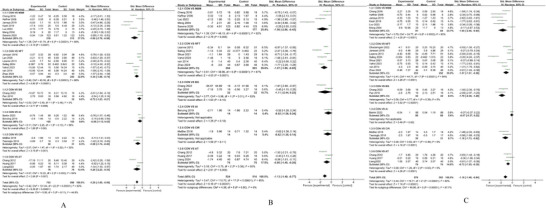
Forest plot of outcomes, A = inhibitory control, B = executive function C = gross motor Skills.

We conducted a pairwise meta‐analysis as part of the NMA and generated forest plots to illustrate the effects of various exercise modalities on the development of Inhibitory Control (Figure 4A), Executive Function (Figure 4B), and Gross Motor Skills (Figure 4C).

In the domain of inhibitory control, the pairwise meta‐analysis revealed that four exercise interventions significantly improved outcomes in children with ADHD compared to the control group. MBM demonstrated the largest effect size (SMD = –1.89, 95% CI [–2.79, –0.98], *p* < 0.0001, *I^2^* = 90%), though with considerable heterogeneity. NFT (SMD = –1.34, 95% CI [–1.88, –0.79], *p* < 0.00001, *I^2^* = 86%), AT (SMD = –1.29, 95% CI [–2.23, –0.35], *p* = 0.007, *I^2^* = 84%), and CIR (SMD = –1.08, 95% CI [–1.74, –0.42], *p* = 0.001, *I^2^* = 32%) also showed substantial benefits. BG exhibited a more modest but still significant effect (SMD = –0.72, 95% CI [–1.23, –0.21], *p* = 0.006, *I^2^* = 0%), while AI did not reach statistical significance (SMD = –0.55, 95% CI [–1.12, 0.02], *p* = 0.06, *I^2^* = 56%).

Regarding executive function, direct pairwise comparisons revealed significant improvements for several interventions. MBM demonstrated the strongest effect (SMD = –1.56, 95% CI [–2.66, ‐0.45], *p* = 0.006), despite considerable heterogeneity (*I^2^* = 92%). It was followed by NFT (SMD = –1.27, 95% CI [–1.89, ‐0.65], *p* < 0.0001, *I^2^* = 87%) and AT (SMD = ‐0.80, 95% CI [–1.40, –0.20], *p* = 0.009, *I^2^* = 65%). In contrast, BG, AI, and CIR did not show statistically significant benefits on executive function in these direct comparisons.

For gross motor skills, five of the six interventions showed significant improvements. BG was the most effective (SMD = –1.79, 95% CI [–2.39, –1.20], *p* < 0.00001, *I^2^* = 0%), followed closely by MBM (SMD = –1.65, 95% CI [–2.46, –0.84], *p* < 0.0001, *I^2^* = 80%) and CIR (SMD = –1.18, 95% CI [–1.73, ‐0.62], *p* < 0.0001, *I^2^* = 0%). NFT (SMD = –1.07, 95% CI [–1.57, –0.58], *p* < 0.0001, *I^2^* = 84%) and AT (SMD = –0.72, 95% CI [–1.05, –0.39], *p* < 0.0001, *I^2^* = 0%) also demonstrated significant benefits, while AI (SMD = –0.07, 95% CI [–0.37, 0.22], *p* = 0.63) remained the only intervention without a statistically significant effect.

### 3.5 NMA

#### 3.5.1 Network Diagram of Included Studies

**FIGURE 5:**
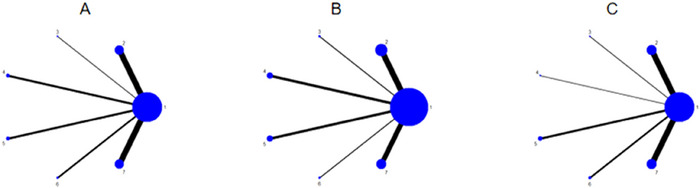
Network plot of outcome indicators, A = inhibitory control B = executive function C = gross motor skills, 1 = CON 2 = MBM 3 = BG 4 = AI 5 = AT 6 = CIR 7 = NFT.

Each dot in the figure represents 1 intervention, for a total of 6 interventions. The straight lines between the dots represent the existence of direct comparisons between interventions, and the thickness of the line represents the number of direct comparisons between the two interventions. Inhibitory control, executive function, and gross motor skills outcome metrics are all 7 interventions (including the control group). The experimental group includes MBM, BG, AI, AT, CIR, and NFT, and the control group is a non‐intervention group. Among these, MBM and NFT were the most widely studied interventions, while BG was investigated in fewer studies. The network diagram of the outcome indicators is detailed in Figure 5.

#### 3.5.2 NMA Matrix of Outcome

Table 3 presents the NMA Matrix of Outcome for inhibitory control, executive function, and gross motor skills.

The ranking probabilities presented in Table 3 are derived from the integrated analysis of all direct and indirect comparisons in the network. Therefore, they provide a holistic perspective on the relative performance of each intervention, which may differ from the conclusions drawn from individual pairwise comparisons in the forest plots due to the contribution of indirect evidence.

For inhibitory control, MBM (SMD = –2.26, 95% CI [–2.97, –1.55]), AT (SMD = –1.22, 95% CI [–2.24, –0.20]), and NFT (SMD = –0.85, 95% CI [–1.50, –0.20]) demonstrated statistically significant effects versus CON. However, CIR (SMD = –1.08, 95% CI [–2.36, 0.20]), AI (SMD = –0.91, 95% CI [–2.71, 0.89]), and BG (SMD = –0.54, 95% CI [–2.33, 1.24]) showed non‐significant effects compared to CON.

For executive function, MBM (SMD = –1.68, 95% CI [–2.47, –0.88]) and NFT (SMD = –1.12, 95% CI [–1.94, –0.30]) exhibited statistically significant improvements relative to CON. In contrast, AI (SMD = –0.98, 95% CI [–2.04, 0.07]), CIR (SMD = –0.91, 95% CI [–2.79, 0.97]), AT (SMD = –0.82, 95% CI [–1.88, 0.24]), and BG (SMD = –0.45, 95% CI [–2.32, 1.42]) demonstrated non‐significant effects versus CON.

For gross motor skills, BG (SMD = –2.08, 95% CI [–3.70, –0.47]) and MBM (SMD = –1.59, 95% CI [–2.20, –0.98]) were the top‐performing interventions, demonstrating the largest and statistically significant effect sizes versus CON. CIR (SMD = –1.21, 95% CI [–2.31, –0.10]), NFT (SMD = –0.55, 95% CI [–1.10, –0.01]), and AI (SMD = –1.20, 95% CI [–2.05, –0.35]) also showed significant benefits relative to CON. In contrast, AT (SMD = –0.68, 95% CI [–1.53, 0.18]) did not show a statistically significant difference compared to CON.

#### 3.5.3 Ranking of Intervention Effectiveness of the Six Exercise Modalities

**FIGURE 6:**
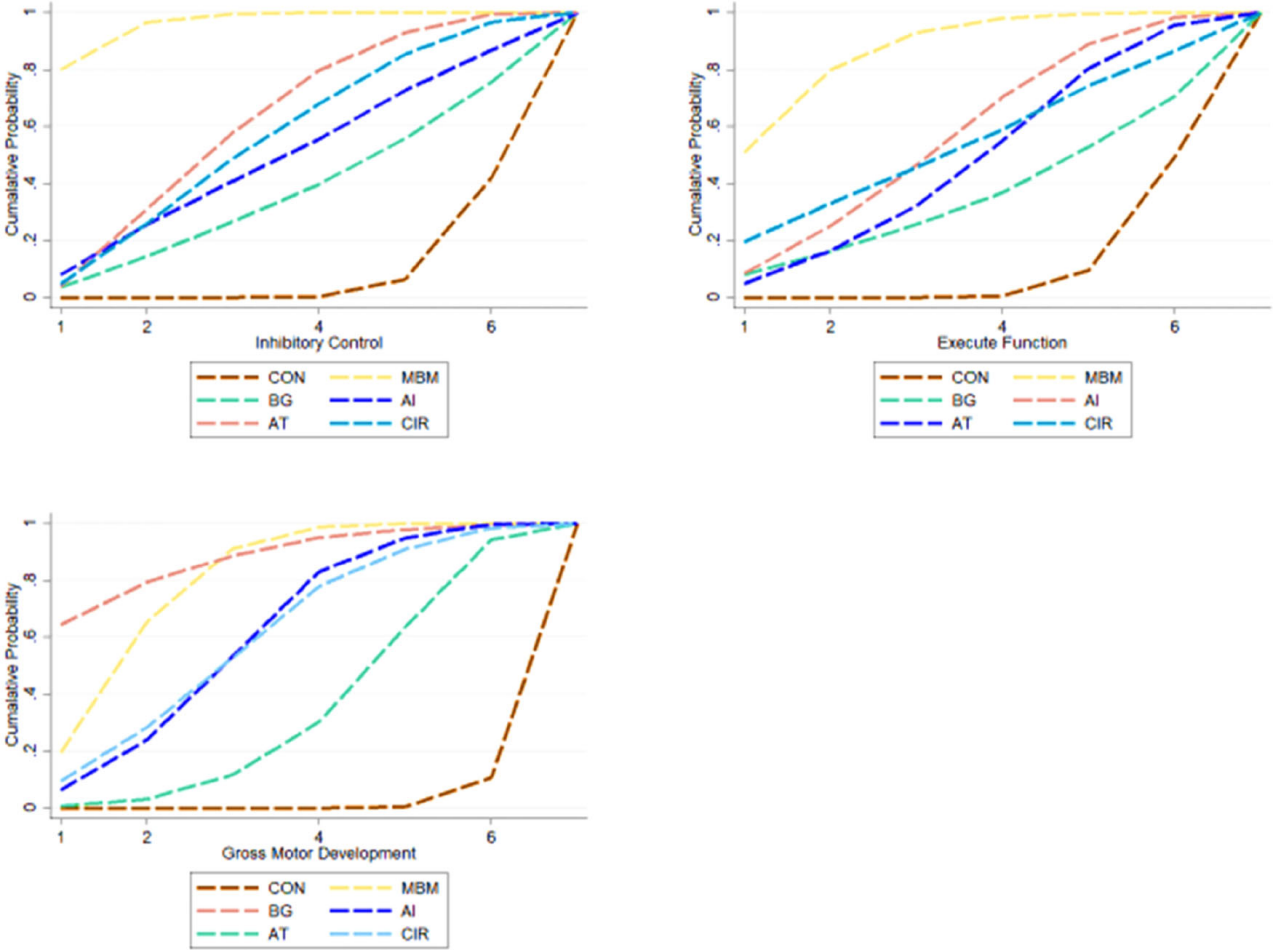
Ranking of intervention effects for outcome indicators.

The ranking of the intervention effects of the six exercise modes is shown in Figure 6 and Table 4.

**TABLE 4:** Ranking of the probability of improving gross motor skills, inhibitory control, and executive function in Children with ADHD by six non‐invasive treatments.

Treatment	Gross motor skills		Inhibitory control		Executive function	
	SUCRA%	rank	SUCRA%	rank	SUCRA%	rank
CON	1.9	7	8.1	7	10.0	7
MBM	79.1	2	96.0	1	86.9	1
BG	87.4	1	35.9	6	34.6	6
AI	60.2	3	48.2	4	56.2	3
AT	33.9	5	60.8	2	47.8	5
CIR	59.6	4	54.9	3	52.1	4
NFT	28.1	6	46.2	5	62.1	2

Inhibitory control indicator: MBM was identified as the most effective intervention for enhancing inhibitory control, substantially outperforming other modalities. AT, CIR, and AI demonstrated intermediate effectiveness, whereas NFT and BG showed more modest benefits compared to the active interventions.

Executive function metrics: MBM consistently ranked highest for improving executive function, followed by NFT. AI and CIR provided moderate improvement, while AT and BG appeared less effective relative to the top‐ranked interventions.

Gross motor skills indicator: Among the six exercise intervention modalities, BG and MBM were ranked as the most effective interventions for improving gross motor skills in children with ADHD, followed by AI and CIR. NFT showed moderate effects, while AT was the least effective.

### 3.6 Small Sample Effect or Publication Bias Tests

**FIGURE 7:**
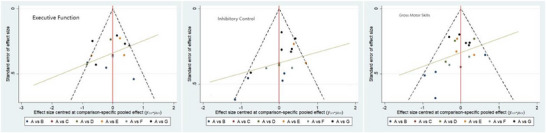
Corrected comparison funnel plot for outcome indicators, A = CON; B = MBM; C = BG; D = AT; E = AI; F = CIR; and G=NFT.

For studies included in the NMA, small sample effect estimates and publication bias tests were performed using corrected‐comparison funnel plots. The included studies were largely symmetrical, suggesting that there was no small‐sample effect in the current study, and no significant publication bias was found. See Figure 7.

## 4 Discussion

### 4.1 Effects of Six Different Exercise Interventions on Inhibitory Control

The results of the NMA (Table 3) indicate that among the six interventions, MBM ranked first in improving inhibitory control in children with ADHD, demonstrating a statistically significant effect. This finding is consistent with previous research (Liang et al. 2024). The efficacy of MBM can be attributed to its multi‐targeted, synergistic nature. Studies suggest that practices such as yoga and tai chi require sustained attention to posture, movement, and breathing, thereby promoting activation of the prefrontal cortex and enhancing functional connectivity within the prefrontal‐striatal neural network, which systematically strengthens inhibitory control (Diamond and Ling 2016).

AT ranked second in improving inhibitory control and also showed a significant effect, aligning with prior evidence. Its mechanism lies in the targeted modulation of core neurological deficits in ADHD. Research indicates that activities such as jogging or swimming effectively enhance neural activity in the prefrontal cortex and anterior cingulate cortex, leading to improved behavioral performance in inhibitory control tasks among children with ADHD (Choi et al. 2015).

AI also demonstrated significant efficacy, which is consistent with the findings of Kollins (Zhang et al. 2018). That study confirmed that FDA‐approved, AI‐driven digital therapeutics can effectively improve inhibitory control. The underlying mechanism involves AI systems dynamically adjusting task difficulty in real time to maintain children within an “optimal challenge zone,” thereby efficiently enhancing inhibitory control abilities (Kollins et al. 2020).

NFT also significantly improved inhibitory control. Evidence suggests that NFT enables children to actively learn and optimize their brain states through real‐time EEG feedback, directly strengthening the regulatory functions of core brain regions involved in inhibitory control, such as the prefrontal cortex and anterior cingulate cortex, thereby enhancing behavioral control (Arns et al. 2009, Gevensleben et al. 2009).

In contrast, CIR did not show a significant effect on inhibitory control, which is consistent with certain earlier studies. The CIR program included in this analysis primarily targeted movement execution, cardiorespiratory fitness, and strength, while lacking sufficient cognitive challenges—such as complex rule adherence, working memory load, or rapid decision‐making. Consequently, its influence on improving inhibitory control in children with ADHD was limited (Benzing et al. 2017).

Similarly, BG did not produce significant effects on inhibitory control, corroborating previous findings. Although BG interventions provide foundational support for brain health and physiological arousal, they do not appear to induce precise activation of neural circuits specifically related to inhibitory control. Compared to more targeted interventions such as NFT and MBM, isolated BG activities may fail to intensively and repeatedly engage higher‐order cognitive processes like response inhibition. Therefore, these results suggest that integrating gross motor activities with cognitively demanding tasks—rather than employing them as standalone cognitive interventions—may represent a more promising direction for future research (Xie et al. 2021).

### 4.2 Effects of Six Different Exercise Interventions on Executive Function

The results of the NMA (Table 3) indicate that among the six interventions, MBM demonstrated significant efficacy in improving executive function in children with ADHD, a finding consistent with previous studies (Zhang et al. 2018). Multiple RCTs and meta‐analyses have confirmed that MBM practices such as yoga and mindfulness effectively alleviate ADHD symptoms and enhance executive function. This efficacy stems from multi‐targeted intervention mechanisms: by integrating physical control, breath regulation, and attentional focus, MBM simultaneously engages core cognitive components, including inhibitory control, working memory, and cognitive flexibility, resulting in sustained and broad improvements (Diamond and Ling 2016).

NFT also showed significant improvements in executive function, aligning with conclusions from several authoritative studies. Multiple meta‐analyses indicate that NFT produces moderate to large effect sizes specifically for symptoms of impulsivity, which reflect underlying deficits in inhibitory control. The mechanism involves NFT guiding children to actively optimize neural activity in core brain regions for inhibitory control—such as the prefrontal cortex and anterior cingulate cortex—through real‐time EEG feedback, thereby strengthening behavioral regulation (Kollins et al. 2020, Arns et al. 2009).

In contrast, AI, CIR, AT, and BG did not demonstrate significant effects on executive function. Although some studies, such as Kollins (Kollins et al. 2020), have reported positive outcomes for AI‐based interventions, systematic reviews highlight substantial individual variability in responsiveness, with long‐term efficacy and generalizability requiring further validation. The limited effects observed for CIR and large‐muscle‐group activities are consistent with conclusions from Benzing (2016) (Gevensleben et al. 2009) and Diamond (2016) (Diamond and Ling 2016), indicating that physical activities lacking integrated cognitive challenges provide limited benefit for executive function improvement in this population.

### 4.3 Effects of Six Different Exercise Interventions on Gross Motor Skills

The results of the NMA (Table 3) indicate that among the six interventions, BG demonstrated a significant effect on improving gross motor skills in children with ADHD, which aligns with previous studies. This efficacy stems from the intervention's direct focus on fundamental movement patterns—such as running, jumping, throwing, and balancing—that closely correspond to the components evaluated in standardized gross motor assessments (Liu et al. 2025).

MBM also showed significant benefits in improving gross motor skills, though this finding diverges from certain earlier reports. For example, Vancampfort (2017) (Damme et al. 2015) noted that while MBM offers certain advantages, targeted large‐muscle‐group training generally produces more comprehensive and pronounced effects in addressing generalized motor delays in children with ADHD. Standardized motor assessment tools such as the BOT‐2 include multidimensional subscales measuring speed, agility, and strength. Although MBM excels in enhancing balance and coordination, its effects on speed, agility, and upper‐limb strength are comparatively limited, which may explain the non‐significant overall score improvements in some studies.

CIR significantly improved gross motor skills, consistent with prior research. Its advantage lies in its comprehensive training model, which systematically challenges strength, endurance, coordination, agility, and balance simultaneously. Through frequent station rotation, CIR provides varied neuromuscular stimulation, thereby supporting holistic gross motor development.

AI also demonstrated significant effects on gross motor skills. This conclusion is based on defining AI intervention as whole‐body exergames utilizing motion‐sensing technology, consistent with related studies. Such interventions, as exemplified by Benzing (2016) (Benzing et al. 2017), are fundamentally AI‐enhanced large‐muscle‐group activities. By using immersive scenarios and real‐time feedback, they motivate children to engage in repetitive, coordinated whole‐body movements, effectively improving strength, coordination, and balance.

NFT significantly enhanced gross motor skills, a finding consistent with the theoretical framework of Gevensleben (2009) (Gevensleben et al. 2009). The underlying rationale is that neuroplasticity changes induced by NFT may generalize beyond cognitive domains, indirectly improving motor performance.

In contrast, AT did not show a significant effect on gross motor skills. Related research (Liu et al. 2025) suggests that the efficacy of physical activity in enhancing motor skills is highly activity‐dependent, with programs incorporating specific skill practice—such as running, jumping, and throwing—producing the most substantial gains. This indicates that AT focused exclusively on cardiorespiratory fitness is less effective than multimodal skill‐based interventions in improving complex motor abilities.

### 4.4 Clinical Implications and Future Directions

A consistent and key finding across all three outcome domains was that while SUCRA rankings suggested a hierarchy of intervention efficacy—such as MBM for inhibitory control and executive function, and BG for gross motor skills—the league table derived from the NMA showed no statistically significant differences in most direct comparisons between active interventions. This discrepancy highlights an important clinical insight: although some interventions may appear preferable, current evidence is insufficient to establish their superiority over other effective modalities.

Rather than limiting choices, this finding enhances the flexibility for designing personalized intervention strategies for children with ADHD. For example, children with prominent hyperactive‐impulsive symptoms may benefit from high‐energy activities such as BG or CIR, which help channel excess energy and improve self‐control. In contrast, those with primarily inattentive symptoms may respond better to MBM or NFT, which enhance cognitive engagement and self‐regulation. The availability of multiple effective options increases the likelihood of identifying activities aligned with a child's interests—a crucial factor for promoting long‐term adherence.

From an implementation perspective, these interventions can be integrated into existing school‐ or community‐based programs, offering scalable and non‐pharmacological complements to conventional behavioral therapy and medication.

Therefore, future research should move beyond the question of “which intervention is best” on average. Instead, the focus should shift toward developing evidence‐based guidelines for matching specific intervention types to individual patient characteristics, such as symptom profile, comorbidities, and preferences. Furthermore, exploring the combined effects of exercise with other treatment modalities in real‐world settings, as well as conducting high‐quality RCTs that directly compare the highest‐ranked interventions (e.g., MBM vs. BG), are essential next steps to refine clinical practice.

When interpreting the findings of this study, it is important to note that the significance of certain interventions differs between traditional pairwise comparisons (forest plots) and NMA (results matrix). This phenomenon is an inherent characteristic of NMA, stemming from the differing evidence bases relied upon by the two analytical methods. Binary comparisons rely solely on direct evidence between a specific intervention and its control group, whereas NMA integrates both direct and indirect evidence across all interventions, forming a unified evidence network. When direct evidence for a specific intervention is relatively sparse or imprecise within the overall network, the NMA model statistically adjusts its effect size toward the network‐wide “mean,” yielding a more conservative yet holistically consistent estimate. Therefore, the results matrix from the NMA (Table 3) provides more robust comparative findings based on all available evidence and should be regarded as the primary basis for this study's conclusions. The forest plot (Figure 4) serves as a supplementary display of direct evidence, with its limitations addressed in the Limitations section of this paper.

The findings of this NMA should be interpreted considering the heterogeneity in the evidence base across interventions. While modalities like MBM are supported by a substantial number of studies, others such as BG and CIR are informed by a smaller, and thus less precise, body of evidence. This heterogeneity does not diminish the potential value of these less‐studied interventions but indicates that their current effect estimates are less stable. Consequently, their ranking should be viewed as preliminary, highlighting a clear need for more high‐quality, focused research to confirm their efficacy and optimal application within personalized intervention strategies for children with ADHD.

### 4.5 Limitation

This study has several limitations. First, despite comprehensive searches in major English and Chinese databases, the exclusion of non‐core journals, non‐English literature (e.g., Spanish, French), and grey literature may affect the generalizability of our findings. Second, while funnel plots suggested no major publication bias, the inherent risk of unpublished negative results and inflated effects in small studies persists. Third, significant clinical and methodological heterogeneity across the included studies (e.g., in intervention protocols and settings) may affect the comparability of the pooled results. This is particularly relevant for interventions with fewer studies (e.g., BG, CIR), where limited evidence reduces the precision and stability of their effect estimates. Finally, our analysis was constrained by the outcome reporting in the original studies. The use of composite scores for executive function prevented a nuanced analysis of its core subcomponents (e.g., working memory, cognitive flexibility), limiting insights into the specific cognitive benefits of different exercises. An important limitation pertains to the interpretation of our findings. As noted, discrepancies in the significance of certain interventions were observed between the conventional pairwise meta‐analysis (Figure 4) and the NMA league table (Table 3). This phenomenon primarily stems from the sparsity of the evidence network for some comparisons. The NMA model, by integrating both direct and indirect evidence, applies statistical shrinkage to effect estimates, particularly for interventions with limited or imprecise direct data. This process yields more conservative and coherent estimates across the entire network but can also reduce the statistical significance of interventions that appeared effective in isolated, direct comparisons. Consequently, while the NMA provides the most comprehensive relative ranking, the findings for less densely connected interventions should be interpreted with caution, as their true effect may be less stable and more susceptible to the incorporation of new evidence. Furthermore, the interpretability of our findings is limited by the heterogeneity among studies and the small number of trials for certain interventions, such as BG and CIR. The limited evidence for these modalities reduces the precision and stability of their estimated effects, meaning their rankings are more uncertain and should be interpreted with caution.

## 5 Conclusion

This study systematically evaluated the effects of six exercise interventions on inhibitory control, executive function, and gross motor skills in children with ADHD using NMA. Results indicate that exercise interventions are generally beneficial for children with ADHD, but the effects are specific: MBM demonstrated the strongest effect in enhancing inhibitory control and executive function (highest SUCRA score) and also significantly improved gross motor skills. NFT showed significant improvements across all three domains. AT yielded significant gains only in inhibitory control, with no significant effects on executive function or gross motor skills. BG and AI produced significant improvements only in gross motor skills, with no statistically significant effects on inhibitory control or executive function. CIR showed only a marginally significant improvement in gross motor skills.

## Author Contributions

**Juan Ouyang**: Conceptualization, methodology, formal analysis, investigation, writing – original draft, visualization. **Yimin Hu**: Data curation, software, validation, writing – original draft, resources. **Yi Xia**: Investigation, formal analysis, writing – review and editing, project administration. **Yi Sheng**: Supervision, funding Acquisition, methodology, writing – review and Editing, validation.

## Funding

This work supported by Shanghai Key Lab of Human Performance (Shanghai University of sport) NO.11DZ2261100.

## Ethics Statement

The authors have nothing to report.

## Consent

The authors have nothing to report.

## Conflicts of Interest

The authors declare no conflicts of interest.

## Data Availability

The data that support the findings of this study are available from the corresponding author upon reasonable request.
